# Kimura disease: a rare cause of a recurrent cheek mass in a Jamaican man

**DOI:** 10.1093/jscr/rjab100

**Published:** 2021-04-20

**Authors:** Geoffrey Williams, Carlos Neblett, Jade Arscott, Sheena McLean, Shereika Warren, Garfield Blake

**Affiliations:** 1 Division of Plastic & Reconstructive Surgery, Department of Surgery, Cornwall Regional Hospital, Montego Bay, Jamaica; 2 Department of Pathology, Cornwall Regional Hospital, Montego Bay, Jamaica

## Abstract

Kimura disease (KD) is a chronic, inflammatory, benign disorder endemic to Asia that typically manifests as a triad of painless masses in the head and neck region, elevated eosinophils and serum immunoglobulin. It usually affects young men in their second and third decades of life and is rarely seen outside of the orient. This is a report of a case of KD in a young man of African descent who presented with a cheek mass. KD was not included in our differential diagnosis, and this report highlights the need to consider this entity, which can be easily missed due to its rarity in the Western world. There is no cure for the disease, and management includes medical and surgical modalities, but local recurrence or relapse is not uncommon.

## INTRODUCTION

Kimura disease (KD) is a rare benign, chronic, inflammatory disorder for which the aetiology is largely unknown [1]. It most commonly presents as painless lymphadenopathy and/or subcutaneous masses in the head and neck region in young males of Asian descent [2]. Rare and sporadic cases of KD have been reported in the Western hemisphere [3]. The authors herein discuss a case of this entity that was managed at a hospital, a regional tertiary referral centre for Plastic and Reconstructive Surgery in the Caribbean. This is the first such published report of a case in Jamaica and adds to the very few reported cases of this condition in persons of African descent [4]. KD was not considered as a part of the differential diagnosis in this case, and the diagnosis was made on histological findings.

## CASE PRESENTATION

A 29-year-old male of African descent presented to the Plastic and Reconstructive Surgery Outpatient Department at a hospital, with a 3-year history of a non-tender, slow-growing swelling to the right cheek. He reported no constitutional symptoms, and his previous medical history was unremarkable.

On examination, there was a 3.0 × 3.0 cm spherical, firm, smooth, non-tender mass on the right cheek. Clinically, the mass appeared confined to the subcutaneous plane with no involvement of the overlying skin or mucosa, and there was no associated lymphadenopathy.

**Figure 1:**
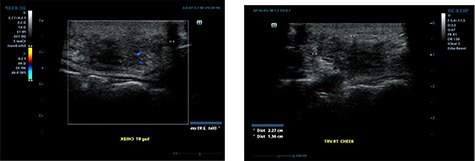
Ultrasound examination demonstrating a heterogenous lesion measuring 2.9 × 2.3 × 1.4 cm mass.

Ultrasound examination revealed a heterogenous lesion measuring 2.9 × 2.3 × 1.4 cm, with associated non-specific ipsilateral Level II cervical lymph nodes (Fig. 1). Haematological test results showed a leukocyte count of 8.47 × 10^9^/l with an eosinophil rate of 17.4% (normal range is 1–5%) and normal renal function.

**Figure 2:**
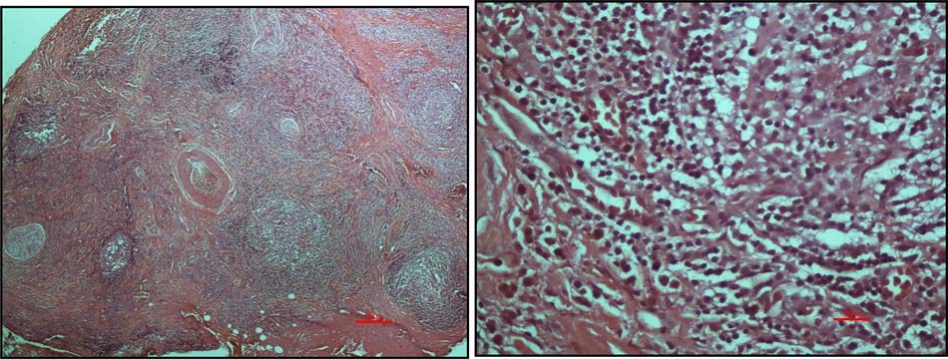
Haematoxylin and eosin-stained micrographs of the mass depicting reactive lymphoid follicles in a fibrous stroma magnification ×4 (**A**) and eosinophilic infiltration at magnification ×40 (**B**).

An excisional biopsy was performed, and the histopathological findings were those of numerous, variably sized lymphoid follicles set within a background of hyalinized fibrous and adipose tissues as well as germinal centres bordered by well-defined mantle zones and numerous eosinophils located in the inter-follicular areas, all in keeping with a diagnosis of KD (Fig. 2). The resection margins were involved but the patient defaulted from the clinic.

**Figure 3:**
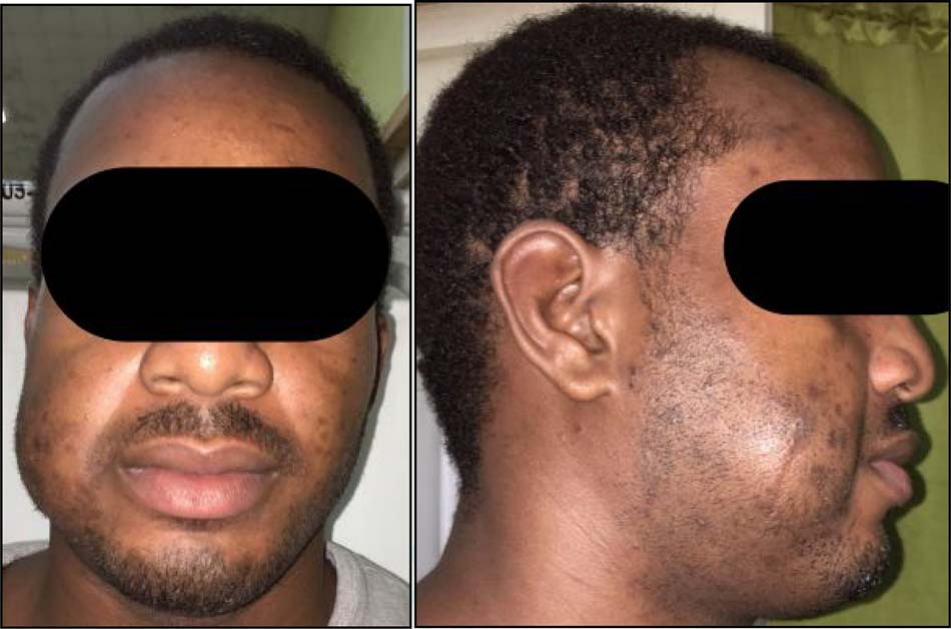
Anterior (**A**) and right lateral (**B**) photographic views depicting the diffuse 5.0 × 5.0 cm swelling to the right cheek (surgical scar from initial excision highlighted) in this Jamaican man.

**Figure 4:**
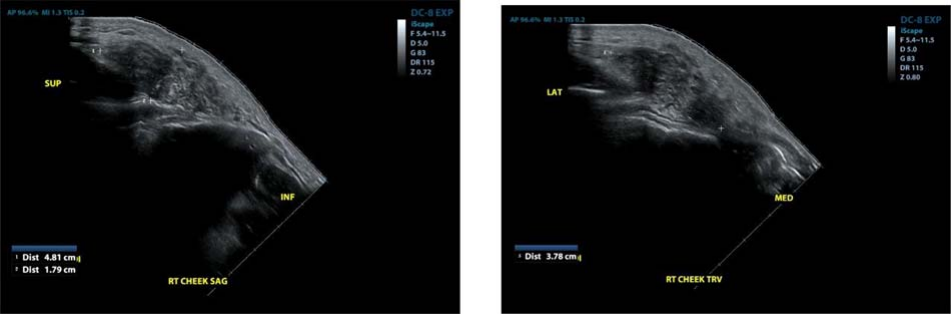
Ultrasound examination demonstrating a heterogenous lesion measuring 4.8 × 3.8 × 1.8 cm mass.

He re-presented 2 years later with a larger 5.0 × 5.0 cm swelling in the same area of the right check (Fig. 3). Ultrasound evaluation demonstrated a 4.8 × 3.8 × 1.8 cm mass with similar features to his previous study (Fig. 4). Blood studies on this occasion showed a leucocytosis with an elevated eosinophil rate of 27.8%. A diagnosis of recurrent KD was made, and this was confirmed on histopathological examination of the re-excised tissue, which again showed involved margins (Fig. 2). The patient was advised to undergo radiation therapy but he refused.

He is now 14 months postoperative since re-excision and has shown no signs of recurrence. He is being seen in the outpatient department on a 3-monthly basis.

## DISCUSSION

KD was first reported by Kimm and Szeto in 1937 in China as ‘eosinophilic hyperplastic lymphogranuloma’, but the details of the pathological features of ‘unusual granulation combined with hyperplastic changes in lymphoid tissues’ were subsequently described by Kimura in 1948 in Japan [1, 5, 6]. This disease process is classically characterized by a triad of painless subcutaneous masses in the head and neck region, blood and tissue eosinophilia and elevated serum immunoglobulin E levels [5]. The latter was not assessed in the index patient. These nodular lesions are usually located in the pre-auricular region, parotid glands and submandibular region along with the larynx and oral cavity of the head and neck with co-existing cervical lymphadenopathy in 30–40% of cases [1]. KD may also manifest with progressive renal impairment with glomerulonephritis or nephrotic syndrome in up to 60% of patients [7].

### Radiological and histopathological appraisal of KD

Classical investigation includes the use of ultrasonography, computed tomography or magnetic resonance imaging to delineate the anatomical structures as well as to rule out more sinister conditions, e.g. tuberculosis, lymphoma, salivary gland cancers, all of which may mimic KD.

In addition, these studies can facilitate image-guided biopsy of the lesion or associated lymph nodes in equivocal cases where tissue histopathology is essential in making the diagnosis [1, 6].

Histologically, KD is identified by the presence of intact lymph node architecture with reactive and prominent germinal centres with dense eosinophilic infiltration of the interfollicular areas along with lysis of the follicles—all of which were present in this case (Fig. 2). Tissue fibrosis, sclerosis, occasional micro-abscesses and vascular proliferation may also be present. Immunofluorescence, not available in our country, confirms the presence of heavy immunoglobulin E (IgE) and variable amounts of IgG, IgM and fibrinogen in the germinal centres [1].

### Principles of KD management

There is no universally accepted standard of management of KD, and there is no cure. Various treatment modalities have been described and include medical management, surgical intervention, radiotherapy and chemotherapy [6].

Medical management mainly consists of continuous low-dose steroids and, less commonly, the use of immune modulators [1]. However, it is worth highlighting that all these conservative strategies are associated with lesion relapse upon discontinuation of the medications [1].

Surgical excision alone is associated with recurrence rates of up to 40%, due to the ill-defined borders of these infiltrative lesions. Furthermore, given the benign nature of the disease, there is always the balance between the removal of involved tissue against the aesthetic and functional deficits, which may result from wide local excision [5].

The use of radiation therapy alone has been shown to have local recurrence rates of up to 75%; however, when used as an adjunct to surgery, recurrence rates are significantly less when compared with either treatment modality as a single entity, and it is now generally accepted that surgery followed by low dose radiation offers the longest periods of remission [5, 6, 8].

KD is a rare entity in the Western world and particularly among persons of African descent, hereby, making the diagnosis exceedingly difficult in our setting.

## CONFLICT OF INTEREST STATEMENT

None declared.

## FUNDING

None.
